# Feasibility of the Implementation of a Technic of Extra-Corporeal Co2 Removal (ECCO2R) in An Intensive Care Unit Which Doesn'T Use Ecmo and Its Real Utilization

**DOI:** 10.1186/2197-425X-3-S1-A505

**Published:** 2015-10-01

**Authors:** V Amilien, JP Ponthus, P Ngasseu, E Barsam, P Lehericey, M Tchir, E Bezian, JF Georger

**Affiliations:** Centre Hospitalier Intercommunal de Villeneuve Saint Georges, Reanimation Polyvalente, Villeneuve Saint Georges, France

## Introduction

The utility and feasibility of ECCO2R in an intensive care unit (ICU) which doesn't use ECMO has not been described. in our general ICU of 15 beds, we don't use ECMO and we decided to introduce ECCO2R in COPD and ARDS patients.

## Objectives

The purpose is to describe the feasibility of this technique in our ICU and the actual uses of it.

## Materials and Method

We chose ILA ACTIVVE® device (NOVALUNG®) with MINILUNG® membrane and a double line femoral catheter (NOVAPORT TWIN 24F). a training for each member of the team was conducted (physicians and nurses) and we wrote a detailed procedure between February 2014 and July 2014. We wrote a procedure for: the preparation of the device, laying the catheter, monitoring, removal of the catheter and the machine, any malfunctions ...). Between august 2014 and February 2015, data were collected for each patient receiving ECCO2R. Indication, mortality at J28, duration of ECCO2R, complications and success of the technique were evaluated.

## Results

During this period, 10 of the 348 patients admitted to our ICU have received ECCO2R therapy. We included 5 patients with ARDS (we laid the device because tidal volume (Vt) was 6ml/kg PBW with a PaO2/FiO2 ratio between 80 and 150, after at least one prone position session, and with a plateau pressure between 25 and 30cm of water and high level of PaCO2). We included 3 COPD patients in failure of non-invasive ventilation (NIV) in a hypercapnic coma, 1 asthmatic patient in severe respiratory acidosis under invasive ventilation (IV) and 1 severe COPD patient unweaning of the IV. Patients with ARDS normalized arterial pH, we decreased Vt (3-4 ml/kg) and respiratory rate. Three of them are alive at J28 and had received 5, 7 and 14 days of ECCO2R and 2 of them died under ECCO2R after 16 and 9 days of ECCO2R. the reason of the death was nosocomial lung infection. None of the COPD patients with hypercapnic coma were intubated, they received 5, 3, 6 days of ECCO2R and were alive at J28. the asthmatic patient received 7 days of ECCO2R, was extubated under ECCO2R and was alive at J28. the unweaning COPD patient was extubated 2 times, reintubated 2 times due to severe neuropathy and tracheal inhalation. He was tracheotomised and weaned from the ECCO2R after 19 days, he was alive at J28 but died at J. There were no bleeding leading by catheter but one hemothorax requiring a blood transfusion during an overdosage of heparin.

## Conclusions

The ECCO2R in a general ICU could have multiple indications in COPD and ARDS patients. These indications remain to be validated but ECCO2R could improve the management of those patients. the implementation of ECCO2R is possible but requires a great investment, a training of the medical and the paramedical team and a written detailed procedure.

